# Molecular detection and genotyping of *Enterocytozoon bieneusi* in pet dogs in Xinjiang, Northwestern China

**DOI:** 10.1051/parasite/2021057

**Published:** 2021-07-20

**Authors:** Yangwenna Cao, Qinglin Tong, Chenhao Zhao, Aikebaierjiang Maimaiti, Liwen Chuai, Junjie Wang, Dingyun Ma, Meng Qi

**Affiliations:** College of Animal Science, Tarim University Alar Xinjiang 843300 PR China

**Keywords:** *Enterocytozoon bieneusi*, Pet dogs, Genotype, Infection rate

## Abstract

*Enterocytozoon bieneusi* is an obligate intracellular parasitic fungi that infects a wide range of mammalian hosts. However, the literature is lacking information regarding the presence and diversity of *E. bieneusi* genotypes in domesticated dogs in Northwestern China. Fecal samples from 604 pet dogs were obtained in 5 cities (Urumqi, Korla, Hotan, Aksu, and Shihezi) in Xinjiang. Screening for *E. bieneusi* was performed, and isolates were genotyped via nested-PCR amplification of the internal transcribed spacer (ITS) of nuclear ribosomal DNA. The infection rate of *E. bieneusi* was 6.3% (38/604). The prevalence of *E. bieneusi* infections in adult animals (>1 year, 10.3%, 15/145) was higher than that in younger (≤1 year) dogs (5.0%, 23/459), which was statistically significant (*p* = 0.021). No significant difference was observed between the different collection sites or between sexes. Eight distinct genotypes were identified, including 5 known genotypes (PtEb IX, EbpC, D, CD9, and Type IV) and 3 novel genotypes (CD11, CD12, CD13). The most prevalent was genotype PtEb IX, being observed in 50.0% (19/38) of the samples, followed by EbpC (31.6%, 12/38), D (5.3%, 2/38), and the remaining genotypes (CD9, Type IV, CD11, CD12, and CD13) were observed in 1 sample (2.6%, 1/38) each. These findings suggest that genotypes PtEb IX and CD9 are canine host-adapted, and likely pose little risk of zoonotic transmission. Moreover, known zoonotic genotypes EbpC, D, and Type IV represent a public health concern and should undergo further molecular epidemiological investigation.

## Abstract (fr)

*Enterocytozoon bieneusi* est un champignon intracellulaire, parasite obligatoire, qui infecte un large éventail d’hôtes mammifères. Cependant, la littérature manque d’informations concernant la présence et la diversité des génotypes d’*E. bieneusi* chez les chiens domestiques dans le nord-ouest de la Chine. Des échantillons fécaux de 604 chiens de compagnie ont été obtenus dans 5 villes (Urumqi, Korla, Hotan, Aksu et Shihezi) au Xinjiang. Le dépistage d’*E. bieneusi* a été effectué et les isolats ont été génotypés par amplification PCR nichée de l’espaceur transcrit interne (ITS) de l’ADN ribosomique nucléaire. Le taux d’infection d’*E. bieneusi* était de 6,3 % (38/604). La prévalence des infections à *E. bieneusi* chez l’adulte (> 1 an, 10,3 %, 15/145) était plus élevée que chez les chiens plus jeunes (≤ 1 an) (5,0 %, 23/459), ce qui était statistiquement significatif (*p* = 0,021). Aucune différence significative n’a été observée entre les différents sites de collecte ou entre les sexes. Huit génotypes distincts ont été identifiés, dont 5 connus (PtEb IX, EbpC, D, CD9, Type IV) et 3 nouveaux (CD11, CD12, CD13). Le plus répandu était le génotype PtEb IX, observé dans 50,0 % (19/38) des échantillons, suivi par EbpC (31,6 %, 12/38), D (5,3 %, 2/38) et les génotypes restants (CD9, Type IV, CD11, CD12 et CD13) ont été observés dans 1 échantillon (2,6 %, 1/38) chacun. Ces résultats suggèrent que les génotypes PtEb IX et CD9 sont adaptés à l’hôte canin et présentent probablement un faible risque de transmission zoonotique. De plus, les génotypes zoonotiques connus EbpC, D et Type IV représentent un problème de santé publique et nécessitent une enquête épidémiologique moléculaire plus approfondie.

## Introduction

Microsporidia are diverse and ubiquitous obligate intracellular parasitic fungi, with diverse hosts ranging from protists to vertebrates, up to and including humans [16, 27]. There are nearly 1500 described microsporidian species in over 200 genera [13]. Of the 17 microsporidian species known to be human pathogens, *Enterocytozoon bieneusi* is the most prevalent one that infects the gastrointestinal tract and is responsible for 90% of human microsporidiosis cases reported globally [16].

First detected in 1985, *E. bieneusi* was isolated from a Haitian AIDS patient suffering from severe diarrhea [8]. As an emerging infectious agent, *E. bieneusi* infection is characterized by acute or chronic diarrhea, malabsorption, and/or wasting [17, 26]. Additionally, immunocompetent individuals with asymptomatic infections are also commonly reported worldwide [23, 27]. Furthermore, *E. bieneusi* has also been detected in animals. Some zoonotic genotypes are commonly identified both in humans and synanthropic animals, which suggests a potential for zoonotic transmission [13].

Due to the small size of its spores and the uncharacteristic staining properties of this organism, it is difficult to detect *E. bieneusi* by routine microscopy. As such, PCR is currently the most reliable tool for diagnosis of *E. bieneusi* infections [13]. Currently, amplification and sequencing of the ribosomal internal transcribed spacer (ITS) is widely used to identify and genotype *E. bieneusi* strains [13]. To date, over 500 *E. bieneusi* genotypes have been defined, which constitute 11 phylogenetic groups with distinct differences in their host specificity and zoonotic potential [11–13, 16]. Group 1 and Group 2 include most of the potentially zoonotic genotypes, whereas the remaining clusters exhibit strong host specificity [16].

The rate of pet ownership is increasing globally, as animals enrich the lives of humans. In China, it has been estimated that approximately 17% of households own companion animals, dogs being the most common numbering 62 million in 2019 (https://www.chyxx.com/industry/202006/874331.html). Although there are benefits of animal companionship, pet animals can carry diseases that may be transmitted to humans, leading to a potential threat to public health. Reports have documented the prevalence and genotype distributions of *E. bieneusi* in dogs worldwide, and the prevalence ranges from 0.8% to 25.8% (Table 2). In China, there are also some reports of the occurrence of *E. bieneusi* in dogs is the east of the country [15, 28, 30, 31]. However, data from Northwestern China are lacking. Therefore, this study aimed to investigate the occurrence and genetic diversity of *E. bieneusi* in pet dogs in the Xinjiang Uygur Autonomous Region (hereinafter referred to as Xinjiang), Northwest China, and to assess the zoonotic potential of any detected *E. bieneusi* strains.

## Materials and methods

### Ethics statement

This study was conducted in accordance with the Chinese Laboratory Animal Administration Act (1988). The Ethics Committee of Tarim University (protocol number: DW201802003) reviewed and approved this sampling protocol. Appropriate permissions and assistance were obtained from the directors or the owners at each pet hospital, shop, or kennel, before collecting fecal samples from the dogs.

### Sample collection and DNA extraction

A total of 604 individual fecal samples were obtained from dogs in 5 cities (Urumqi, Korla, Hotan, Aksu, and Shihezi) in Xinjiang, China, between April 2018 and June 2020. Within the collection sites, 3 types of sample sources were included: 8 pet hospitals, 17 pet shops, and 6 kennels. The age of sampled pet dogs ranged from 2 months to 13 years.

The samples were collected directly from the rectum of each animal or immediately after defecation and picked up using sterile disposable gloves. Identification number, collection site, sample source, sex, and age were recorded with the help of the site directors or dog owners. The stools were non-diarrheal at the time of sampling. All samples were placed on ice in separate containers and transported to the laboratory immediately. Prior to DNA extraction, fecal samples were stored at 4 °C.

### DNA extraction

Total genomic DNA was extracted from each fecal sample using an E.Z.N.A.^®^ Stool DNA kit (D4015-02, Omega Biotek Inc., Norcross, GA, USA). Briefly, about 200 mg of each fecal sample were placed in a 2 mL centrifuge tube containing 200 mg of glass beads and placed on ice. Next, 300 μL of buffer SP1 and proteinase K were added, and the tubes were incubated at 70 °C for 10 min. Subsequently, all the procedures outlined in product manual were performed according to the kit manufacturer’s instructions. Finally, DNA was eluted in 200 μL of elution buffer and the extract was stored at −20 °C until used in PCR amplifications.

### Nested PCR amplification

*Enterocytozoon bieneusi* was identified via nested-PCR amplification of a ~390 bp region of the internal transcribed spacer (ITS) of nuclear ribosomal DNA for each sample [4]. The nested-PCR primers EBITS3: 5′ – GGTCATAGGGATGAAGAG – 3′ and EBITS4: 5′ – TTCGAGTTCTTTCGCGCTC – 3′ were used for the external reaction, and EBITS1: 5′ – GCTCTGAATATCTATGGCT – 3′ and EBITS2.4: 5′ – ATCGCCGACGGATCCAAGTG – 3′ for the internal reaction. PCR cycling conditions were an external cycle of 94 °C for 5 min, followed by 35 cycles of 94 °C for 30 s, 57 °C for 30 s (55 °C for the internal reaction), and 72 °C for 40 s, and a final extension of 72 °C for 8 min. 2 × EasyTaq PCR SuperMix (TransGene Biotech Co., Beijing, China) were used for each PCR amplification. To ensure accuracy and rule out contamination, DNA from dog-derived genotype D was used as the positive control, and distilled water devoid of DNA as the negative control in each PCR.

### Sequencing phylogenetic analyses

Positive secondary PCR amplicons were sequenced by a commercial sequencing company (GENEWIZ, Suzhou, China). The sequence accuracy was confirmed via bidirectional sequencing, and the sequences obtained were aligned using ClustalX 2.1 (http://www.clustal.org/) with reference sequences downloaded from GenBank (https://www.ncbi.nlm.nih.gov/genbank/) to determine the species and genotypes. Only the ITS region should be considered when designating the new *E. bieneusi* genotypes [13]. Representative sequences of the isolated genotypes were submitted to GenBank at the National Center for Biotechnology Information under the following accession numbers: MW412816–MW412818.

Bayesian inference (BI) and Monte Carlo Markov chain methods were used to construct the phylogenetic trees in MrBayes (version 3.2.6) (http://nbisweden.github.io/MrBayes/). The posterior probability values were calculated by running 1,000,000 generations. A 50% majority-rule consensus tree was constructed from the final 75% of the trees generated via BI. Analyses were run 3 times to ensure convergence and insensitivity to priors.

### Statistical analysis

All statistical analyses were performed using SPSS 22.0 software. In the univariate analyses, a Fisher’s exact test was used to compare the prevalence of the *E. bieneusi* infections in groups constructed according to collection sites, sample source, sex, and age. The infection rates between groups of various sources were compared using a chi-square test. Significant differences were accepted when the *p*-value was <0.05.

## Results

### Prevalence of *E. bieneusi* in pet dogs

Among the collected 604 fecal samples collected from pet dogs, 6.3% (38/604) were positive for *E. bieneusi*. Specifically, a higher prevalence of *E. bieneusi* infections was observed in Aksu (13.6%, 2/22), Shihezi (12.2%, 5/41), and Urumqi (9.9%, 18/191). Lower prevalence was observed in Korla (3.6%, 5/137) and Hotan (3.3%, 7/213). Among the collection sites, the prevalences of *E. bieneusi* infections from pet dogs in Korla (*χ*^2^ = 4.080, *p* = 0.043) and Hotan (*χ*^2^ = 6.535, *p* = 0.011) were statistically lower than the other cities investigated (Table 1).

**Table 1:** Distributions of *E. bieneusi* in pet dogs from Xinjiang, China.

Category	No. sampled	No. Positive	Infection [95% CI]	*χ*^2^-value, *p*-value	*Enterocytozoon bieneusi* genotypes (no.)
Collection site					
Urumqi	191	18	9.4% [5.2–13.6]		EbpC (9), PtEb IX (5), D (2), CD9 (1), CD11 (1)
Korla	137	5	3.6% [0.5–6.8]	4.080, 0.043	PtEb IX (2), EbpC (2), CD12 (1)
Hotan	213	7	3.3% [0.9–5.7]	6.535, 0.011	PtEb IX (5), Type IV (1), CD13 (1)
Aksu	22	3	13.6% [0–29.2]	0.394, 0.530	PtEb IX (2), EbpC (1)
Shihezi	41	5	12.2% [1.7–22.7]	0.290, 0.590	PtEb IX (5)
Sample source					
Pet hospital	134	9	6.7% [2.4–11.0]		EbpC (5), D (2), PtEb IX (1), CD11 (1)
Pet shop	256	16	6.3% [3.3–9.2]	0.032, 0.858	PtEb IX (7), EbpC (5), CD9 (1), Type IV (1), CD12 (1), CD13 (1)
Pet kennel	214	13	6.1% [2.8–9.3]	0.057, 0.811	PtEb IX (11), EbpC (2)
Sex					
Female	326	24	7.4% [4.5–10.2]		PtEb IX (12), EbpC (6), D (2), CD9 (1), Type IV (1), CD11 (1), CD12 (1)
Male	278	14	5.0% [2.4–7.6]	1.377, 0.241	PtEb IX (7), EbpC (6), CD13 (1)
Age					
≤1 year	459	23	5.0% [3.0–7.0]		PtEb IX (8), EbpC (7), D (2), CD9 (1), Type IV (1), CD11 (1), New2 (1), CD13 (1)
>1 year	145	15	10.3% [5.3–15.4]	5.318, 0.021	PtEb IX (10), EbpC (5)
Total	604	38	6.3% [4.3–8.2]		PtEb IX (19), EbpC (12), D (2), CD9 (1), Type IV (1), CD11 (1), CD12 (1), CD13 (1)

### *Enterocytozoon bieneusi* infections by sample source, sex, and age group

Different rates of *E. bieneusi* infections in pet dogs were observed in different groups (sample source, sex, and age) in the present study. For the different sample sources, 3 sample sources were involved. The prevalences of *E. bieneusi* infections in pet hospitals, pet shops, and pet kennels were 6.7% (9/134), 6.3% (16/256), and 6.1% (13/214), respectively. There were no statistically significant differences observed among the different collection sites (*χ*^2^ = 0.057, *p* = 0.811). For sex, although the female animals (7.4%, 24/326) were slightly higher than males (5.0%, 14/278), the differences were not statistically significant (*χ*^2^ = 1.377, *p* = 0.241). For age groups, adult (>1 year) dogs (10.3%, 15/145) were infected at a higher rate than were juvenile (≤1 year) dogs (5.0%, 23/459) (*χ*^2^ = 5.318, *p* = 0.021, Table 1).

### Genotype distributions and sequence analysis of *E. bieneusi*

The sequence analysis of 38 positive samples revealed the presence of eight different genotypes in pet dogs in Xinjiang, China. Five of them (PtEb IX, EbpC, D, CD9, and Type IV) were known genotypes and 3 (CD11, CD12, CD13) were novel genotypes. The most prevalent genotype was PtEb IX, observed in 50.0% (19/38) of samples, followed by EbpC (31.6%, 12/38), and D (5.3%, 2/38). The remaining genotypes (CD9, Type IV, CD11, CD12, CD13) were each observed in one (2.6%, 1/38) sample (Table 1). No mixed infections of *E. bieneusi* genotypes were identified in the present study.

Genotype PtEb IX was identical to an isolate from dogs (KJ668719) in China. Genotype EbpC was identical to an isolate from dogs (MN902235) in China. Genotype D was identical to a human isolate (MN136771) from China. Genotype Type IV was identical to a Chinese isolate from a hedgehog (MK841506).

The novel genotypes CD11 (D559) and CD12 (D756) had 99.74% and 99.23% homology to the previously isolate identified in a wild boar (MK681466), respectively. These two isolates had 1 and 3 substitutions to isolate MK681466, at 110 (T → C), and 106 (T → C), 141 (A → G), and 169 (T → C), respectively. The novel genotype CD13 (D1338) had 99.74% homology to fox isolate MN029060 in China, with one substitution at 162 (T → C).

### Phylogenetic analyses

**Figure 1:**
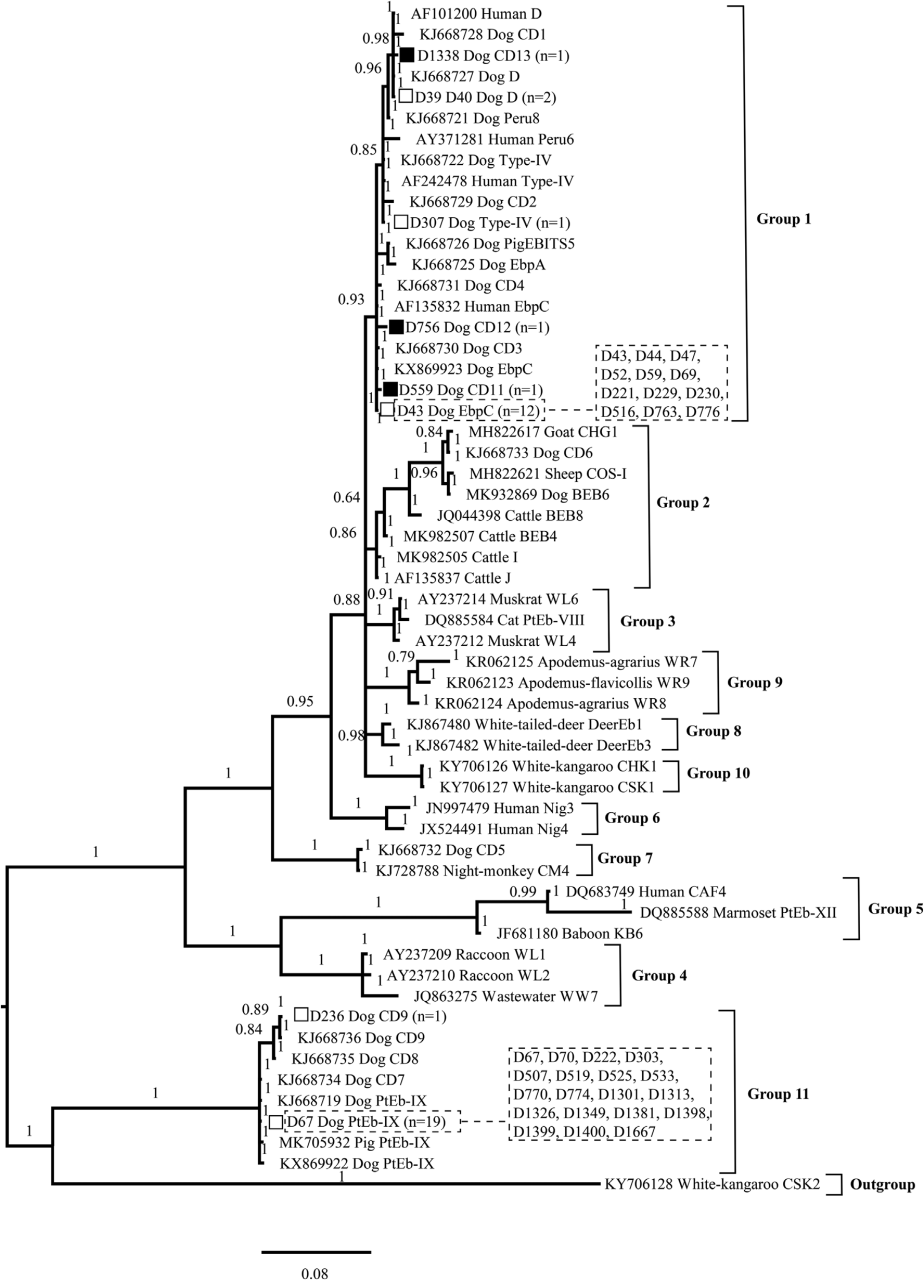
Phylogenetic tree based on Bayesian inference (BI) analysis of the *Enterocytozoon bieneusi* ITS sequences. Statistically significant posterior probabilities (>0.7) are indicated on the branches. Known and novel *E. bieneusi* ITS genotypes identified in the present study are indicated by empty and filled squares, respectively.

Bayesian inference phylogenetic analysis revealed that the 3 known (EbpC, D, and Type IV) genotypes and 3 novel (CD11, CD12, CD13) genotypes identified herein clustered into Group 1, which suggests zoonotic potential. The other genotypes PtEb IX and CD9 were previously assigned to Group 11, which tends to exhibit host specificity (Fig. 1).

## Discussion

In the present study, a 6.3% (38/604) *E. bieneusi* positivity rate among pet dogs in Xinjiang, China was observed. A significantly higher infection rate was observed in pet dogs (22.9%, 149/651) in Guangdong, China [28], and pet and stray dogs (15.5%, 54/348) in China in another study [11], domestic dogs (11.7%, 2/17; 9.6%, 7/73) in 2 studies in Spain [9, 18], and stray dogs (15.0%, 18/120) in Colombia [25]. Significantly lower infection rates were reported in owned and sheltered dogs (0.8%, 2/237) in Northern Spain [5], family pet dogs (4.4%, 26/597) in Japan [21], and domestic dogs (4.4%, 15/342) in Australia [32]. However, similar results were reported in pet dogs (6.0%, 29/485) in Shanghai, China [30], and in pet and stray dogs (6.7%, 18/267) in Heilongjiang, China [14].

Among the facility types (pet hospital, pet shop, and pet kennel) and sexes (female and male) from which fecal samples were collected in the present study, no differences in *E. bieneusi* positivity were observed (Table 1). For the age groups, the prevalence of *E. bieneusi* infections in adult (>1 year) dogs (10.3%, 15/145) was higher than that in juveniles (≤1 year, 5.0%, 23/459). This observation was consistent with previous reports in pet and stray dogs (10.1% vs. 1.8%) in Heilongjiang, China [14], pet and stray dogs (12.8% vs. 6.1%) throughout China [11], and stray dogs (18.8% vs. 0) in Colombia [25]. However, the prevalence cannot be compared due to the differences in study population composition of age, region, and living conditions between the previous and present studies. In general, the prevalence reported in these studies may be attributable to differences in the geographic area, feeding sites, life-style, age distribution, sample sizes, animals health status, management systems, and population densities of the animals tested, as well as other unidentified factors.

Eight different *E. bieneusi* ITS genotypes were identified in 38 positive pet dogs in the present study. Genotype PtEb IX was dominant and the most prevalent genotype identified in pet dogs in the present study and in many previous investigations (Table 2). Genotype PtEb IX was primarily isolated from dogs worldwide, and cats in China and Australia, and a wild badger in Spain [11, 24, 32]. Genotype PtEb IX is considered to be a canine host-adapted genotype [16, 20]. Genotypes EbpC and D were identified in 12 and 2 samples, respectively. Both genotypes have been widely identified in humans, non-human primates, and pigs, with occasional reports in dogs, horses, and wildlife in China [29]. Genotype Type IV, identified in one dog sample here, has previously been reported in humans, non-human primates, and wild animals in China [29]. Taken together, the data suggest that these three genotypes have the potential for zoonotic transmission. Since *E. bieneusi* was mainly transmitted by the fecal-oral route, the assessment of possible sources had been focused on exposure of pet dogs to contaminated soil or water while taking part in outdoor activities. This was partly confirmed in previous studies [5, 22, 28, 30]. Genotype CD9 has previously been identified in one female pet dog sample in Xi’an, Shaanxi Province, China [11]. The remaining 3 novel genotypes (CD11, CD12, CD13), as well as 3 known (EbpC, D, and Type IV) genotypes identified herein clustered into the zoonotic potential Group 1, based on the BI phylogenetic analysis. The other genotypes, PtEb IX and CD9, clustered into Group 11, which indicates some host specificity (Fig. 1). There are several reports of *E. bieneusi* in humans, farm animals, and wild animals in Xinjiang, China (Supplemental Material Table S1). Identification of the same *E. bieneusi* genotypes between humans and animals, and different types of animals, indicates the potential likelihood that these genotypes are mutual transmitted between these hosts.

**Table 2:** Prevalence and genotype distributions of *E. bieneusi* in dogs.

Collection site	Sample source	Infection (no. positive/sampled)	Genotype distributions (no.)	References
Australia (Victoria)	Domestic dog	4.4% (15/342)	**PtEb IX** (9), D (5), VIC_dog1 (1)	[32]
China (Heilongjiang)	Pet and stray dog	6.7% (18/267)	**PtEb IX (12)**, EbpC (1), D (1), NED2 (1), EbpC/NED1 (1), PtEb IX/NED3 (1), PtEb IX/NED4 (1)	[14]
China (Changchun)	Pet market dogs	7.7% (2/26)	CHN5 (1), CHN6 (1)	[31]
China (Shanghai)	Household, pet shops, veterinary clinic dogs	6.0% (29/485)	**PtEb IX (28)**, D (1)	[30]
China (Guangzhou)	Pets dogs	22.9% (149/651)	**PtEb IX (56)**, GD1 (38), GD2 (24), D (12), EbpC (7), CD9 (4), I (2), GD3 (2), GD4 (2), GD5 (1), GD6 (1)	[28]
China (Anhui, Zhejiang)	Veterinary hospitals dogs	8.6% (27/315)	**PtEb IX(16)**, EbpC(4), CHD 3(3), CHD1(2), CHD2 (2)	[15]
China (Henan, Sichuan, Shaanxi, Chongqing)	Pet and stray dogs	15.5% (54/348)	**PtEb IX (26)**, CD8 (4), O (4), D (3), CD7 (2), EbpA (2), CM1 (2), EbpC (1), Peru8 (1), type IV (1), PigEBITS5 (1), CD1 (1), CD2 (1), CD3 (1), CD4 (1), CD5 (1), CD6 (1), CD9 (1)	[11]
Colombia (Bogota)	Stray dogs	15.0% (18/120)	**PtEb IX (16)**, type IV (1), Peru 5/WL11 (1)	[25]
Egypt (Giza)	Domestic dogs	13.0% (14/108)	N/A	[2]
Iran (Tehran)	Stray dogs	5.3% (4/75)	**D (4)**	[7]
Iran (Ardebil)	Ownership dogs	11.8% (2/17)	N/A	[3]
Iran (Isfahan)	Animal clinic dogs	25.8% (8/100)	N/A	[10]
Japan (Osaka, Ishikawa, Niigata)	Pet and stray dogs	2.5% (2/79)	**PtEb IX (2)**	[1]
Japan	Family pet dogs	4.4% (26/597)	**PtEb IX (26)**	[21]
Poland	Household dogs	4.9% (4/82)	PtEb IX (2), D (2)	[22]
Portugal (Lisbon)	Pet owners and shelters dogs	3^a^	PtEb IX (1), Peru 6 (1); D/Peru 9 (1)	[17]
Spain (Galicia)	Domestic dogs	11.8% (2/17)	N/A	[18]
Spain	Domestic dogs	9.6% (7/73)	**A (7)**	[9]
Spain (Madrid)	Domestic dogs	8.7% (4/46)	N/A	[6]
Spain (Álava)	Owned and sheltered dogs	0.8% (2/237)	PtEb IX (1), BEB6 (1)	[5]
Switzerland	Farm dogs	8.3% (3/36)	**PtEb IX (3)**	[19]

N/A, not available; Bold = dominant genotype.

a This article only reports the genetic diversity of samples previously identified as *E. bieneusi-*positive.

## Conclusions

The results presented here show that the prevalence of *E. bieneusi* in pet dogs in Xinjiang was relatively low. Present and previous studies indicated that genotypes PtEb IX and CD9 were considered host-adapted genotypes and are unlikely to exhibit zoonotic transmission. Genotypes EbpC, D, and Type IV, and 3 novel genotypes (CD11, CD12, and CD13) possibly exhibit zoonotic transmission potential. To determine the actual threat that these genotypes pose to public health requires further investigation.

## Supplemental material

Supplementary material is available at https://www.parasite-journal.org/10.1051/parasite/2021057/olm *Table S1*. Previous reports of *Enterocytozoon bieneusi* in humans, farm animals, and wild animals in Xinjiang, China.

## Conflict of interest

The authors declare that they have no conflict of interest.

## References

[1] Abe N, Kimata I, Iseki M. 2009. Molecular evidence of *Enterocytozoon bieneusi* in Japan. Journal of Veterinary Medical Science, 71(2), 217–219. 10.1292/jvms.71.217 19262036

[2] Al-Herrawy AZ, Gad MA. 2016. Microsporidial spores in fecal samples of some domesticated animals living in Giza, Egypt. Iranian Journal of Parasitology, 11(2), 195–203. 28096853 PMC5236096

[3] Askari Z, Mirjalali H, Mohebali M, Zarei Z, Shojaei S, Rezaeian T, Rezaeian M. 2015. Molecular detection and identification of zoonotic microsporidia spore in fecal samples of some animals with close-contact to human. Iranian Journal of Parasitology, 10(3), 381–388. 26622293 PMC4662738

[4] Buckholt MA, Lee JH, Tzipori S. 2002. Prevalence of *Enterocytozoon bieneusi* in swine: an 18-month survey at a slaughterhouse in Massachusetts. Applied and Environmental Microbiology, 68(5), 2595–2599. 11976142 10.1128/AEM.68.5.2595-2599.2002 PMC127518

[5] Dashti A, Santín M, Cano L, de Lucio A, Bailo B, de Mingo MH, Köster PC, Fernández-Basterra JA, Aramburu-Aguirre J, López-Molina N, Fernández-Crespo JC, Calero-Bernal R, Carmena D. 2019. Occurrence and genetic diversity of *Enterocytozoon bieneusi* (Microsporidia) in owned and sheltered dogs and cats in Northern Spain. Parasitology Research, 118(10), 2979–2987. 31435764 10.1007/s00436-019-06428-1

[6] del Águila C, Izquierdo F, Navajas R, Pieniazek NJ, Miró G, Alonso AI, Da Silva AJ, Fenoy S. 1999. *Enterocytozoon bieneusi* in animals: rabbits and dogs as new hosts. Journal of Eukaryotic Microbiology, 46(5), 8S–9S. 10519225

[7] Delrobaei M, Jamshidi S, Shayan P, Ebrahimzade E, Ashrafi Tamai I, Rezaeian M, Mirjalali H. 2019. Molecular detection and genotyping of intestinal microsporidia from stray dogs in Iran. Iranian Journal of Parasitology, 14(1), 159–166. 31123481 PMC6511603

[8] Desportes I, Le Charpentier Y, Galian A, Bernard F, Cochand-Priollet B, Lavergne A, Ravisse P, Modigliani R. 1985. Occurrence of a new microsporidan: *Enterocytozoon bieneusi* n.g., n. sp., in the enterocytes of a human patient with AIDS. Journal of Protozoology, 32(2), 250–254. 10.1111/j.1550-7408.1985.tb03046.x 4009510

[9] Galván-Díaz AL, Magnet A, Fenoy S, Henriques-Gil N, Haro M, Gordo FP, Millán J, Miró G, del Águila C, Izquierdo F. 2014. Microsporidia detection and genotyping study of human pathogenic *E. bieneusi* in animals from Spain. PLoS One, 9(3), e92289. 24651457 10.1371/journal.pone.0092289 PMC3961313

[10] Jamshidi Sh, Tabrizi AS, Bahrami M, Momtaz H. 2012. Microsporidia in household dogs and cats in Iran; a zoonotic concern. Veterinary Parasitology, 185(2–4), 121–123. 22035849 10.1016/j.vetpar.2011.10.002

[11] Karim MR, Dong H, Yu F, Jian F, Zhang L, Wang R, Zhang S, Rume FI, Ning C, Xiao L. 2014. Genetic diversity in *Enterocytozoon bieneusi* isolates from dogs and cats in China: host specificity and public health implications. Journal of Clinical Microbiology, 52(9), 3297–3302. 24989604 10.1128/JCM.01352-14 PMC4313153

[12] Li J, Jiang Y, Wang W, Chao L, Jia Y, Yuan Y, Wang J, Qiu J, Qi M. 2020. Molecular identification and genotyping of *Enterocytozoon bieneusi* in experimental rats in China. Experimental Parasitology, 210, 107850. 32027893 10.1016/j.exppara.2020.107850

[13] Li W, Feng Y, Xiao L. 2020. Diagnosis and molecular typing of *Enterocytozoon bieneusi*: the significant role of domestic animals in transmission of human microsporidiosis. Research in Veterinary Science, 133, 251–261. 33035931 10.1016/j.rvsc.2020.09.030

[14] Li W, Li Y, Song M, Lu Y, Yang J, Tao W, Jiang Y, Wan Q, Zhang S, Xiao L. 2015. Prevalence and genetic characteristics of *Cryptosporidium*, *Enterocytozoon bieneusi* and *Giardia duodenalis* in cats and dogs in Heilongjiang province, China. Veterinary Parasitology, 208(3–4), 125–134. 25665462 10.1016/j.vetpar.2015.01.014

[15] Li WC, Qin J, Wang K, Gu YF. 2018. Genotypes of *Enterocytozoon bieneusi* in dogs and cats in Eastern China. Iranian Journal of Parasitology, 13(3), 457–465. 30483338 PMC6243171

[16] Li W, Feng Y, Santin M. 2019. Host specifcity of *Enterocytozoon bieneusi* and public health implications. Trends in Parasitology, 35, 436–451. 31076351 10.1016/j.pt.2019.04.004

[17] Lobo ML, Xiao L, Cama V, Stevens T, Antunes F, Matos O. 2006. Genotypes of *Enterocytozoon bieneusi* in mammals in Portugal. Journal of Eukaryotic Microbiology, 53(Suppl 1), S61–64. 10.1111/j.1550-7408.2006.00174.x 17169069

[18] Lores B, del Aguila C, Arias C. 2002. *Enterocytozoon bieneusi* (microsporidia) in faecal samples from domestic animals from Galicia. Spain. Memórias do Instituto Oswaldo Cruz, 97(7), 941–945. 12471418 10.1590/s0074-02762002000700003

[19] Mathis A, Breitenmoser AC, Deplazes P. 1999. Detection of new *Enterocytozoon* genotypes in faecal samples of farm dogs and a cat. Parasite, 6(2), 189–193. 10416194 10.1051/parasite/1999062189

[20] Ou Y, Jiang W, Roellig DM, Wan Z, Li N, Guo Y, Feng Y, Xiao L. 2020. Characterizations of *Enterocytozoon bieneusi* at new genetic loci reveal a lack of strict host specificity among common genotypes and the existence of a canine-adapted *Enterocytozoon* species. International Journal for Parasitology, 51(2–3), 215–223. 33275946 10.1016/j.ijpara.2020.09.008

[21] Phrompraphai T, Itoh N, Iijima Y, Ito Y, Kimura Y. 2019. Molecular detection and genotyping of *Enterocytozoon bieneusi* in family pet dogs obtained from different routes in Japan. Parasitology International, 70, 86–88. 30825524 10.1016/j.parint.2019.02.010

[22] Piekarska J, Kicia M, Wesołowska M, Kopacz Ż, Gorczykowski M, Szczepankiewicz B, Kváč M, Sak B. 2017. Zoonotic microsporidia in dogs and cats in Poland. Veterinary Parasitology, 246, 108–111. 28969771 10.1016/j.vetpar.2017.09.011

[23] Sak B, Brady D, Pelikánová M, Květoňová D, Rost M, Kostka M, Tolarová V, Hůzová Z, Kváč M. 2011. Unapparent microsporidial infection among immunocompetent humans in the Czech Republic. Journal of Clinical Microbiology, 49(3), 1064–1070. 21191056 10.1128/JCM.01147-10 PMC3067711

[24] Santín M, Calero-Bernal R, Carmena D, Mateo M, Balseiro A, Barral M, Lima Barbero JF, Habela MÁ. 2018. Molecular characterization of *Enterocytozoon bieneusi* in wild carnivores in Spain. Journal of Eukaryotic Microbiology, 65(4), 468–474. 10.1111/jeu.12492 29230898

[25] Santín M, Cortés Vecino JA, Fayer R. 2008. *Enterocytozoon bieneusi* genotypes in dogs in Bogota, Colombia. American Journal of Tropical Medicine and Hygiene, 79(2), 215–217. 18689627

[26] Stark D, van Hal S, Barratt J, Ellis J, Marriott D, Harkness J. 2009. Limited genetic diversity among genotypes of *Enterocytozoon bieneusi* strains isolated from HIV-infected patients from Sydney, Australia. Journal of Medical Microbiology, 58(3), 355–357. 19208886 10.1099/jmm.0.006445-0

[27] Stentiford GD, Becnel -J, Weiss LM, Keeling PJ, Didier ES, Williams BP, Bjornson S, Kent ML, Freeman MA, Brown MJF, Troemel ER, Roesel K, Sokolova Y, Snowden KF, Solter L. 2016. Microsporidia – emergent pathogens in the global food chain. Trends in Parasitology, 32, 336–348. 26796229 10.1016/j.pt.2015.12.004 PMC4818719

[28] Wang H, Lin X, Sun Y, Qi N, Lv M, Xiao W, Chen Y, Xiang R, Sun M, Zhang L. 2020. Occurrence, risk factors and genotypes of *Enterocytozoon bieneusi* in dogs and cats in Guangzhou, southern China: high genotype diversity and zoonotic concern. BMC Veterinary Research, 16(1), 201. 32552737 10.1186/s12917-020-02421-4 PMC7301972

[29] Wang SS, Wang RJ, Fan XC, Liu TL, Zhang LX, Zhao GH. 2018. Prevalence and genotypes of *Enterocytozoon bieneusi* in China. Acta Tropica, 183, 142–152. 29660311 10.1016/j.actatropica.2018.04.017

[30] Xu H, Jin Y, Wu W, Li P, Wang L, Li N, Feng Y, Xiao L. 2016. Genotypes of *Cryptosporidium* spp., *Enterocytozoon bieneusi* and *Giardia duodenalis* in dogs and cats in Shanghai, China. Parasites & Vectors, 9, 121. 26932267 10.1186/s13071-016-1409-5 PMC4774012

[31] Zhang X, Wang Z, Su Y, Liang X, Sun X, Peng S, Lu H, Jiang N, Yin J, Xiang M, Chen Q. 2011. Identification and genotyping of *Enterocytozoon bieneusi* in China. Journal of Clinical Microbiology, 49(5), 2006–2008. 21389159 10.1128/JCM.00372-11 PMC3122652

[32] Zhang Y, Koehler AV, Wang T, Cunliffe D, Gasser RB. 2019. *Enterocytozoon bieneusi* genotypes in cats and dogs in Victoria, Australia. BMC Microbiology, 19(1), 183. 31395004 10.1186/s12866-019-1563-y PMC6686557

