# Supplementary material for: Molecular detection and genotyping of Enterocytozoon bieneusi in pet dogs in Xinjiang, Northwestern China
Source: Parasite. 2021 Jul 20;28:57. doi: 10.1051/parasite/2021057 (PMC8290926; doi:10.1051/parasite/2021057)
Supplement: Supplementary file 1 — Table S1. Previous reports of Enterocytozoon bieneusi in humans, farm animals, and wild animals in Xinjiang, China. [file parasite-28-57-s1.pdf]

Table 1S. Previously reports of *Enterocytozoon bieneusi* in humans, farm and wild animals in Xinjiang, China

Host populations	Infection (No. of positive/sampled)	Genotype distributions (no.)	References
Children	5.9% (36/609)	A (3), CHN6 (1), CXJH1 (1), CXJH 2 (1), CXJH 3 (1), D (6), EbpA (3), KB-1 (1), NIA1 (19)	[1]
Rhesus macaque	56.5% (13/23)	CM1 (5), D (2), Mul1 (6)	[2]
Dairy calves	16.5% (85/514)	BEB4 (4), CC4 (1), D (2), EbpC (2), I (19), J (57)	[3]
Dairy calves	52.0% (130/250)	J (108), EbpC (9), PigEBITS5 (5), CHV4 (3), CHC3 (1), CS-9 (1), KIN-1 (1), CH5 (1), CAM5 (1)	[4]
Grazing sheep	6.3% (20/318)	BEB6 (12), CHG1 (1), CHG3 (1), CHS3 (1), CHS8 (1), COS-I (2), XJS1 (1), XJS2 (1)	[5]
Sheep	19.2% (19/99)	BEB6 (18), CHXJS1 (1)	[6]
Pigs	48.6% (389/801)	CHC5 (2), CS-1 (5), CS-4 (20), CS-7 (3), CS-9 (1), D (17), EbpA (129), EbpC (168), EbpD (5), H (2), PigEb4 (12), PigEBITS5 (19), WildBoar8 (3), XJP-II (2), XJP-III (1)	[7]
Rabbits	2.8% (9/321)	J (5), BEB8 (3), Type IV (1)	[8]
Grazing horses	30.9% (81/262)	BEB6 (9), CHG19 (2), CM6 (4), CM7 (2), CM8 (1), CS-1(1), CS-4(1), D (1), EpbA (20), EbpC (21), G (3), horse1 (4), horse2 (2), O (4), Peru8 (1), XJH1 (2), XJH2 (1), XJH3 (1), XJH4 (1)	[9]
Donkeys	2.5% (17/680)	horse1 (5), D (3), NCD-2 (3), BEB6 (2), BEB4 (1), NIAI (1), XJD1 (1), XJD2 (1)	[10]
Alpacas	15.1% (28/185)	ALP1 (53), ALP2 (1), ALP3 (18), ALP4 (1), ALP5 (1), ALP6 (1), ALP7 (2), ALP8 (1), BEB6 (2), CHALT1 (1), D (2), J (2), P (16), Type IV (4)	[11]
Farmed mink	6.1% (65/1071)	CAM5 (1), D (17), EbpA (1), EbpC (13), HLJM-1 (3), HLJM-2 (1), NCM-1 (5), NCM-2 (1), Peru 11 (18), PigEBITS7 (4), XJMI-1 (1)	[12]
Blue foxes	15.7% (100/638)	CHN-DC1 (2), CHN–F1 (1), D (60), EbpC (5), NCF1 (3), NCF2 (13), NCF3 (1), NCF4 (1), NCF5 (2), NCF6 (1), NCF7 (1), Peru8 (4), Type IV (5), WildBoar3 (1)	[12]
Raccoon dogs	12.2% (111/911)	CHN-DC1 (11), CHN–F1 (10), CHN-R1 (1), CHG1 (1), D (32), EbpA (1), NCF2 (33), NCR1 (2), NCR2 (5), Peru8 (3), Type IV (11), WildBoar3 (1)	[12]
Bactrian camels	30.0% (122/407)	CAM1 (72), EbpC (23), CAM2 (8), EbpA (5), CAM4 (5), Henan-IV (1), BEB6(1), CM8 (1), CHG16 (1), O (1), WL17 (1), CAM3 (1), CAM5 (1), CAM6 (1)	[13]
Edible bullfrogs	20.7% (61/295)	EbpC (40), BLC11 (3), BLC1 (1), BLC2 (1), BLC3 (1), BLC4 (1), BLC5 (1), BLC6 (1), BLC7 (1), BLC8 (1), BLC9 (1), BLC10 (1), BLC12 (1), BLC13 (1),	[14]

	BLC14 (1), BLC15 (1), BLC16 (1), BLC17 (1), BLC18 (1), BLC19 (1)

References:

1. Qi M, Yu F, Zhao A, Zhang Y, Wei Z, Li D, Zhang L. Unusual dominant genotype NIA1 of *Enterocytozoon bieneusi* in children in Southern Xinjiang, China. PLoS Negl Trop Dis. 2020; 14(6):e0008293.
2. Yu M, Liu X, Xie M, Li D, Ni Q, Zhang M, Wu J, Xu H, Yao Y. Epidemiological investigation and genotypes of *Enterocytozoon bieneusi* in 11 captive Rhesus macaque populations. Int J Parasitol Parasites Wildl. 2020; 13:191-195.
3. Qi M, Jing B, Jian F, Wang R, Zhang S, Wang H, Ning C, Zhang L. Dominance of *Enterocytozoon bieneusi* genotype J in dairy calves in Xinjiang, Northwest China. Parasitol Int. 2017; 66(1):960-963.
4. Zhao A, Zhang K, Xu C, Wang T, Qi M, Li J. Longitudinal identification of *Enterocytozoon bieneusi* in dairy calves on a farm in Southern Xinjiang, China. Comp Immunol Microbiol Infect Dis. 2020; 73:101550.
5. Qi M, Zhang Z, Zhao A, Jing B, Guan G, Luo J, Zhang L. Distribution and molecular characterization of *Cryptosporidium* spp., *Giardia duodenalis*, and *Enterocytozoon bieneusi* amongst grazing adult sheep in Xinjiang, China. Parasitol Int. 2019; 71:80-86.
6. Yang H, Mi R, Cheng L, Huang Y, An R, Zhang Y, Jia H, Zhang X, Wang X, Han X, Chen Z. Prevalence and genetic diversity of *Enterocytozoon bieneusi* in sheep in China. Parasit Vectors. 2018; 11(1):587.
7. Li DF, Zhang Y, Jiang YX, Xing JM, Tao DY, Zhao AY, Cui ZH, Jing B, Qi M, Zhang LX. Genotyping and Zoonotic Potential of Enterocytozoon bieneusi in Pigs in Xinjiang, China. Front Microbiol. 2019; 10:2401.
8. Zhang X, Qi M, Jing B, Yu F, Wu Y, Chang Y, Zhao A, Wei Z, Dong H, Zhang L. Molecular Characterization of *Cryptosporidium* spp., *Giardia duodenalis*, and *Enterocytozoon bieneusi* in Rabbits in Xinjiang, China. J Eukaryot Microbiol. 2018; 65(6):854-859.
9. Qi M, Wang R, Wang H, Jian F, Li J, Zhao J, Dong H, Zhu H, Ning C, Zhang L.

Enterocytozoon bieneusi genotypes in grazing horses in China and their zoonotic transmission potential. J Eukaryot Microbiol. 2016; 63(5):591-7.

10. Zhao A, Zhang Y, Wang W, Jing B, Xing J, Tao D, Zhao W, Qi M. *Enterocytozoon bieneusi* in donkeys from Xinjiang, China: prevalence, molecular characterization and the assessment of zoonotic risk. BMC Vet Res. 2020; 16(1):196.
11. Zhang Q, Wang H, Zhao A, Zhao W, Wei Z, Li Z, Qi M. Molecular detection of *Enterocytozoon bieneusi* in alpacas (*Vicugna pacos*) in Xinjiang, China. Parasite. 2019; 26:31.
12. Zhang Y, Xin L, Zhao A, Xu C, Wang T, Jing B, Qi M. Molecular detection and genotypes of *Enterocytozoon bieneusi* in farmed mink (*Neovison vison*), blue foxes (*Alopex lagopus*), and raccoon dogs (*Nyctereutes procyonoides*) in Xinjiang, China. Int J Parasitol Parasites Wildl. 2021; 14:211-215.
13. Qi M, Li J, Zhao A, Cui Z, Wei Z, Jing B, Zhang L. Host specificity of *Enterocytozoon bieneusi* genotypes in Bactrian camels (*Camelus bactrianus*) in China. Parasit Vectors. 2018; 11(1):219.
14. Ding H, Zhao A, Wang L, Gao N, Sun Y, Li J, Qi M. Genotypes and zoonotic potential of *Enterocytozoon bieneusi* in edible bullfrogs (*Lithobates catesbeiana*) in China. Int J Parasitol Parasites Wildl. 2020; 11:103-107.
